# Impact of cerebral blood flow and amyloid load on SUVR bias

**DOI:** 10.1186/s13550-022-00898-8

**Published:** 2022-05-12

**Authors:** Fiona Heeman, Maqsood Yaqub, Janine Hendriks, Bart N. M. van Berckel, Lyduine E. Collij, Katherine R. Gray, Richard Manber, Robin Wolz, Valentina Garibotto, Catriona Wimberley, Craig Ritchie, Frederik Barkhof, Juan Domingo Gispert, David Vállez García, Isadora Lopes Alves, Adriaan A. Lammertsma, on behalf of the AMYPAD Consortium

**Affiliations:** 1 Amsterdam UMC, Vrije Universiteit Amsterdam, Radiology and Nuclear Medicine, Amsterdam Neuroscience, De Boelelaan 1117, 1081 HV Amsterdam, The Netherlands; 2 IXICO Plc, London, UK; 3 NIMTLab, Faculty of Medicine, Geneva University, Geneva, Switzerland; 4 Division of Nuclear Medicine and Molecular Imaging, Geneva University Hospitals, Geneva, Switzerland; 5 Edinburgh Imaging, Queen’s Medical Research Institute, University of Edinburgh, Edinburgh, UK; 6 UCL, Institutes of Neurology and Healthcare Engineering, London, UK; 7 Barcelonaβeta Brain Research Centre, Pasqual Maragall Foundation, Barcelona, Spain; 8 Centro de Investigación Biomédica en Red de Bioingeniería, Biomateriales y Nanomedicina (CIBER-BBN), Madrid, Spain; 9 Department of Experimental and Health Sciences, Universitat Pompeu Fabra, Barcelona, Spain; 10 IMIM (Hospital del Mar Medical Research Institute), Barcelona, Spain

**Keywords:** Alzheimer’s disease, Amyloid PET, Cerebral blood flow, Quantification, SUVR bias

## Abstract

**Background:**

Despite its widespread use, the semi-quantitative standardized uptake value ratio (SUVR) may be biased compared with the distribution volume ratio (DVR). This bias may be partially explained by changes in cerebral blood flow (CBF) and is likely to be also dependent on the extent of the underlying amyloid-β (Aβ) burden. This study aimed to compare SUVR with DVR and to evaluate the effects of underlying Aβ burden and CBF on bias in SUVR in mainly cognitively unimpaired participants. Participants were scanned according to a dual-time window protocol, with either [^18^F]flutemetamol (*N* = 90) or [^18^F]florbetaben (*N* = 31). The validated basisfunction-based implementation of the two-step simplified reference tissue model was used to derive DVR and *R*_1_ parametric images, and SUVR was calculated from 90 to 110 min post-injection, all with the cerebellar grey matter as reference tissue. First, linear regression and Bland–Altman analyses were used to compare (regional) SUVR with DVR. Then, generalized linear models were applied to evaluate whether (bias in) SUVR relative to DVR could be explained by *R*_1_ for the global cortical average (GCA), precuneus, posterior cingulate, and orbitofrontal region.

**Results:**

Despite high correlations (GCA: *R*^2^ ≥ 0.85), large overestimation and proportional bias of SUVR relative to DVR was observed. Negative associations were observed between both SUVR or SUVR_bias_ and *R*_1,_ albeit non-significant.

**Conclusion:**

The present findings demonstrate that bias in SUVR relative to DVR is strongly related to underlying Aβ burden. Furthermore, in a cohort consisting mainly of cognitively unimpaired individuals, the effect of relative CBF on bias in SUVR appears limited. EudraCT Number: 2018-002277-22, registered on: 25-06-2018.

**Supplementary Information:**

The online version contains supplementary material available at 10.1186/s13550-022-00898-8.

## Background

At present, amyloid positron emission tomography (PET) imaging is routinely used in specialized memory clinics to support the diagnosis of Alzheimer’s disease (AD). Routinely, a static acquisition is performed and the resulting image is used to visually determine the presence or absence of amyloid-beta (Aβ) plaques by a trained nuclear medicine physician [1, 2]. In addition to their clinical use, static scans have also been widely used for research purposes, mainly because of their technical and logistical simplicity. From these scans, the commonly used standardized uptake value ratio (SUVR) can be calculated, by simply dividing tracer uptake in the target by that in a reference region. Nevertheless, previous studies have demonstrated that SUVR can be biased compared with the non-displaceable binding potential (*BP*_ND_) or distribution volume ratio (DVR = *BP*_ND_ + 1) derived from a dynamic scan [3–5]. In particular, SUVR can be affected by changes in cerebral blood flow (CBF), tracer clearance, and/or extraction fraction [3, 4, 6]. The effects of relative CBF have been illustrated in a longitudinal observational study that reported a decrease in [^11^C]PiB SUVR in a clinical population, while DVR remained unchanged, and accompanying simulations demonstrated that this decrease in SUVR was likely related to a decrease in relative CBF (*R*_1_) [4]. Nonetheless, the explanatory effect of changes in relative CBF on SUVR needs to be further evaluated in larger clinical data sets and for other (amyloid) tracers, given that each tracer has distinct kinetics.

The degree of bias in SUVR also depends on the extent of the Aβ burden [3], and, in the case of AD, it is known that disease stage and Aβ burden are not linearly correlated [7]. Yet, most AD studies have compared SUVR and DVR only at a (diagnostic) group level, and therefore, the relationship between bias and extent of underlying Aβ burden is not fully characterized [5, 8, 9].

Over the last decade, the focus of amyloid PET studies has shifted to the early phases of AD, emphasizing the importance of understanding the bias in SUVR in a pre-dementia group [10–12]. In particular, considering that confounding factors such as changes in CBF, tracer clearance, and extraction fraction may not be constant throughout the disease, their impact on SUVR could also differ in this pre-clinical population compared with AD dementia patients [3, 13, 14].Therefore, the purpose of the present study was to investigate bias in SUVR relative to DVR in elderly individuals and assess to what extent this bias can be explained by underlying Aβ burden and CBF for both [^18^F]flutemetamol and [^18^F]florbetaben.

## Materials and methods

### Participants

The study included *N* = 121 participants from the AMYloid Imaging to Prevent Alzheimer’s Disease (AMYPAD) Prognostic and Natural History Study (PNHS) [15], who were scanned across four different centres: The University of Edinburgh (UEDIN), Barcelona βeta Brain Research Center (BBRC), Amsterdam University Medical Centre, location VUmc (Amsterdam UMC, VUmc), and University of Geneva (UNIGE). All participants had at least one PET and one T1-weighted MR scan available, and visual assessment of the PET scans was performed locally by a trained nuclear medicine physician according to the manufacturer’s reading guidelines [1, 2]. Readers were blinded to the quantitative outcome measure of the scans. In addition, participants underwent standard neurological screening and neuropsychological assessment (e.g. mini-mental state examination, MMSE) and information regarding their *APOE-*ε4 status was available. Before participating in the study, all participants provided written informed consent in accordance with the Declaration of Helsinki. Study protocols were approved by all local Medical Ethics Review Committees. EudraCT Number: 2018-002277-22, registered on: 25-06-2018, https://www.clinicaltrialsregister.eu/ctr-search/trial/2018-002277-22/NL.

### Image acquisition

Prior to each PET scan, an MR sequence (MRAC) or low-dose CT (ldCT) was acquired for attenuation correction purposes (Additional file 1: Table S1 specifies which centres used an MR and which a CT scanner). Participants then received a bolus injection of either [^18^F]flutemetamol **(***N* = 90, 186 ± 10 MBq) or [^18^F]florbetaben (*N* = 31, 283 ± 20 MBq) and underwent a dynamic PET scan according to a dual-time window protocol [16]. This scanning protocol consisted of an early dynamic scan from 0 to 30 min post-injection (p.i.) followed by a break of 60 min, and then a late dynamic scan from 90 to 110 min p.i. For each scanner, recommended clinical reconstruction settings for that scanner were used and scans were reconstructed into 18 frames (6 × 5, 3 × 10, 4 × 60, 2 × 150, 2 × 300, and 1 × 600 s) for the early scan, and four frames (4 × 300 s) for the late scan. All sites applied the following corrections: detector normalization, dead time, attenuation, scatter, decay, and randoms. Site-specific reconstruction methods can be found in Additional file 1: Table S1.

### Image QC and processing

First, a quality control (QC) check of the image meta-data was performed (i.e. frame start times, duration, and the number of frames) and the presence of motion between PET scans, and corresponding MRAC or ldCT, was evaluated. In the case of severe motion (> 5 mm), these scans were excluded from analyses. In addition, it was verified whether the whole brain was in the field of view and whether no technical errors occurred, such as a delay in acquisition start time. Next, presence of between-frame motion was assessed visually. Then, both early and late phases of the PET scan were coregistered to the T1-weighted MR scan using rigid registration and the resulting PET scans were resampled to have the same voxel size as the T1-weighted MR. This was done to prevent changes to the predefined region of interest (ROI) template images, which were already in the same space as the T1-weighted MR scan. Following this step, coregistered PET images were combined and the resulting scan was divided into five blocks of 10 min duration (A: frames 1–15, B: frames 16–17, C: frame 18, D: frames 19–20, and E: frames 21–22). Presence of motion between blocks was assessed visually, by comparing whether contours between blocks were overlapping. Next, using rigid co-registration (based on Elastix software), blocks were co-registered and the registration parameters were saved [17, 18]. Based on a visual assessment and the registration parameters, it was determined whether motion exceeded the maximum allowed threshold (1 mm translation in each direction) and should be corrected for (using Elastix-based rigid co-registration). Subsequently, subject-specific regions of interest generated using Learning Embeddings for Atlas Propagation (LEAP) [19] (in T1-weighted MR space) were applied to the combined PET scan to extract a reference tissue time-activity curve (TAC) of the cerebellar grey matter. As regions of sufficient volume were used (> 5 ml), no partial volume corrections were performed.

### Parametric analysis

Interpolation of the missing data points of the reference tissue TAC (corresponding to the 60 min break) was performed using the reversible two tissue compartment model (4-rate constants) with additional blood volume fraction parameter (2T4k_V_b_) and a tracer-specific plasma input function as described previously [16]. Visual QC of the interpolated reference tissue TAC was performed to assess whether the interpolation of the missing data points had a smooth connection with the measured data. In the case of sub-optimal interpolation (i.e. clear discontinuity between interpolated and measured data), cubic interpolation was used as alternative (*N* = 8). Next, parametric modelling was performed using the basisfunction-based implementation of the two-step simplified reference tissue model (SRTM2) [20], as implemented in the PPET software package [21] and validated previously [22], to compute parametric *BP*_ND_ and relative tracer delivery (*R*_1_) images. For SRTM2, *k*_2_’ was determined by taking the median *k*_2_’ across all voxels with a *BP*_ND_ higher than 0.05 from a first run using receptor parametric mapping (RPM) [23, 24]. In addition, SUVR was calculated from 90 to 110 min p.i. To allow for comparability between metrics, DVR was calculated as *BP*_ND_ + 1. Finally, the subject-specific global cortical average (GCA) template, which is a composite region consisting of frontal, temporal, parietal, and insular cortices, precuneus, and striatum [25], was applied to the parametric data to obtain global SUVR and DVR values. In addition, the subject-specific ROIs generated using LEAP were used to extract values for regions that are known to show early accumulation of Aβ plaques: precuneus, posterior cingulate cortex (PCC), and orbitofrontal cortex (OFC) [12, 26, 27].

### Statistical analysis

All statistical analyses were performed in R (version:4.0.3; R Foundation for Statistical Computing, Vienna, Austria) and stratified per tracer. A statistically significant result was defined as *p* < 0.05; no corrections for multiple comparisons were applied.

First, demographic differences between Aβ-positive and negative participants (based on a visual assessment) were investigated using *t* tests (or in the case of non-normal distribution, Mann–Whitney *U-*tests) and Chi-square tests.

#### Relationship between SUVR and DVR

Linear regression analyses were used to assess the relationships between SUVR and DVR. In addition, Bland–Altman analyses [28] were used to assess potential bias between these metrics, and the presence of proportional bias was determined visually for the GCA and three early regions. When present, proportional bias was further evaluated by fitting a regression line through the Bland–Altman plot.

#### Relationship between (bias in) SUVR and relative CBF

First, bias in SUVR relative to DVR (SUVR_bias_) was calculated ((SUVR-DVR)/DVR*100%). Then, generalized linear models (GLMs) were constructed to understand whether the relative tracer delivery (*R*_1_) could explain the remaining variance of either SUVR or SUVR_bias_ beyond the main predictor, DVR. Note, the covariate “centre” is only included for [^18^F]flutemetamol.

$$\begin{aligned} & {\text{SUVR}} \sim {\text{DVR}} + R_{1} + {\text{DVR}}*R_{1} + {\text{Age}} + {\text{Sex}} + {\text{APOE}} - \varepsilon 4 + {\text{centre}} \\ & {\text{SUVR}}_{{{\text{bias}}}} \sim {\text{DVR}} + R_{1} + {\text{DVR}}*R_{1} + {\text{Age}} + {\text{Sex}} + {\text{APOE}} - \varepsilon 4 + {\text{centre}} \\ \end{aligned}$$

Before applying the GLMs to the data, all predictors (DVR, *R*_1_, and DVR*R_1_) and covariates (age, sex, *APOE-*ε4 carriership, and centre in the case of [^18^F]flutemetamol) were correlated with each other to check for collinearity. This was done using Pearson’s correlation, point-biserial correlation, and Goodman Kruskal’ lambda for continuous, continuous and categorical or categorical variables, respectively. In the case of high overlap (*r* ≥ 0.70) [29], the variables were correlated with the dependent variable (SUVR and SUVR_bias_) to determine which one should be deleted from the model to remove redundancy, i.e. the one with the lowest correlation. All analyses with the final model were performed using the GCA, as well as with the three early regions.

## Results

### Participants

**Fig. 1:**
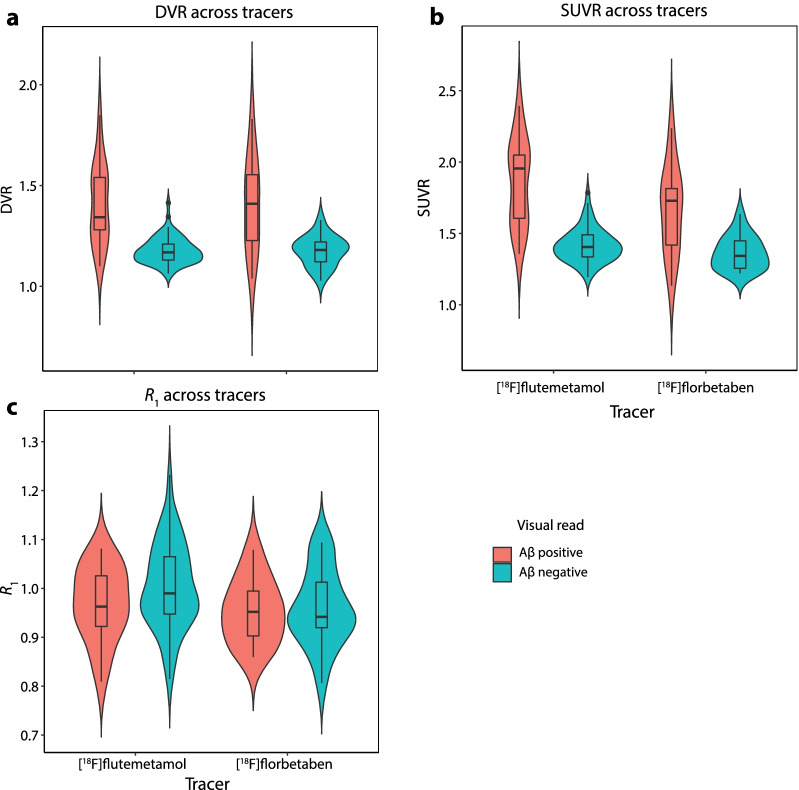
**Parameter distribution across tracers**. Violin plots showing the distribution of **a** DVR, **b** SUVR and **c** *R*_1_ for Aβ positive and negative scans. Small boxplots inside the violin plots display median and quartile range of the distribution

Participant characteristics are shown in Table 1. Most participants were cognitively unimpaired (*N* = 104), while a minority (*N* = 17) showed impairment to some extent, based upon the clinical dementia rating (CDR) score (CDR = 0.5). For participants scanned with [^18^F]flutemetamol, 21.1% (19/90) were visually assessed as Aβ-positive, while for participants scanned with [^18^F]florbetaben, this was 38.7% (12/31). Furthermore, Aβ-positive participants scanned with [^18^F]flutemetamol were slightly older than the Aβ-negative group (*p* < 0.01). As expected, the proportion of *APOE-*ε4 carriers and the overall amyloid burden (in SUVR and DVR units) were higher among Aβ-positive compared with Aβ-negative participants for both tracers, while no differences were observed in *R*_1_ (Fig. 1).

**Table 1:** Participant demographics

	[^18^F]flutemetamol			[^18^F]florbetaben		
	All (*N* = 90)	Aβ-negative (*N* = 71)	Aβ-positive (*N* = 19)	All (*N* = 31)	Aβ-negative (*N* = 19)	Aβ-positive (*N* = 12)
Age	67.2 ± 6.3	66.2 ± 6.3**	70.7 ± 4.7	67.3 ± 7.4	66.0 ± 6.7	69.5 ± 8.2
Females %	54.4	57.7	42.1	67.7	73.7	58.3
MMSE	28.9 ± 1.4	29.2 ± 1.1	28.2 ± 2.1	29.1 ± 1.3	29.4 ± 0.8	28.7 ± 1.8
*APOE-*ε4 + %	41.6	34.3*	68.4	35.5	15.8*	66.7
SUVR	1.51 ± 0.3	1.41 ± 0.1**	1.86 ± 0.3	1.48 ± 0.2	1.36 ± 0.1**	1.66 ± 0.3
DVR	1.23 ± 0.1	1.18 ± 0.1**	1.41 ± 0.2	1.26 ± 0.2	1.18 ± 0.1**	1.40 ± 0.2
*R*_1_	1.00 ± 0.1	1.00 ± 0.1	0.97 ± 0.1	0.96 ± 0.2	0.96 ± 0.1	0.95 ± 0.1
Centre	A,B,C,D	A,B,C,D	A,B,C	A	A	A
Inj. dose (MBq)	186.2 ± 10.4	188.8 ± 10.1	184.0 ± 7.7	282.9 ± 19.7	284.4 ± 17.9	280.5 ± 22.9

Values depicted as mean ± SD, MMSE = mini-mental state examination, Inj. dose = Net injected dose *p < 0.05, **p < 0.01, compared with the Aβ-positive group. A = Amsterdam UMC, B = BBRC, C = UNIGE, D = Edinburgh

#### Relationship between SUVR and DVR

**Fig. 2:**
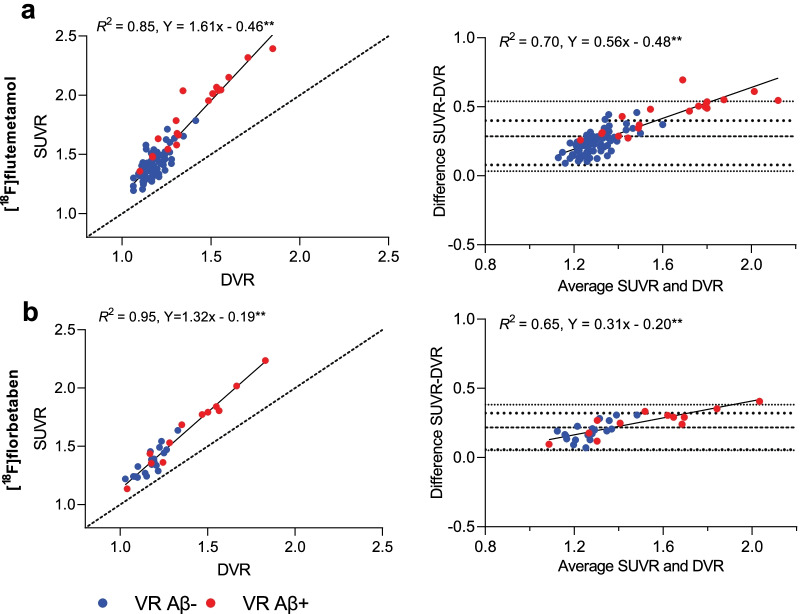
**Relationship between DVR and SUVR.** Correlation and Bland–Altman plots to assess the relationship between global cortical DVR and SUVR for **a** [^18^F]flutemetamol and **b** [^18^F]florbetaben. Dotted lines corresponds to 95% Limits of Agreement. **p < 0.001, VR: visual read

High correlations were observed between global SUVR and DVR ([^18^F]flutemetamol: *R*^2^ = 0.85 [^18^F]florbetaben: *R*^2^ = 0.95, Fig. 2), as for early regions, with small differences between regions ([^18^F]flutemetamol precuneus: *R*^2^ = 0.85, OFC: *R*^2^ = 0.86, PCC: *R*^2^ = 0.82, [^18^F]florbetaben precuneus: *R*^2^ = 0.93, OFC: *R*^2^ = 0.87, PCC: *R*^2^ = 0.85).

SUVR showed an overestimation compared with global DVR for both tracers ([^18^F]flutemetamol mean bias: 0.29, 95% limits of agreement (LoA) 0.03–0.54, and [^18^F]florbetaben mean bias: 0.22 95% LoA 0.05–0.38), which was strongly proportional to the underlying amyloid burden, and slightly larger for [^18^F]flutemetamol ([^18^F]flutemetamol: *R*^2^ = 0.70, slope = 0.56 and intercept = − 0.48, [^18^F]florbetaben: *R*^2^ = 0.65, slope = 0.31 and intercept = − 0.20, Fig. 2). For each of the early regions, a similar pattern in terms of overestimation and proportional bias was observed, although for most early regions, the proportionality was slightly stronger ([^18^F]flutemetamol precuneus: *R*^2^ = 0.81, slope = 0.74 and intercept = − 0.79, OFC: *R*^2^ = 0.74, slope = 0.59 and intercept = − 0.54, PCC: *R*^2^ = 0.73, slope = 0.66 and intercept = − 0.56, and [^18^F]florbetaben precuneus: *R*^2^ = 0.70, slope = 0.39 and intercept = − 0.32, OFC: *R*^2^ = 0.44, slope = 0.31 and intercept = − 0.23, PCC: *R*^2^ = 0.61, slope = 0.48 and intercept = − 0.35).

#### Relationship between independent variables

The univariate correlation analysis between all predictor (DVR, *R*_1_, and DVR*R_1_) and covariate variables (age, sex, *APOE-*ε4 carriership, and centre in the case of [^18^F]flutemetamol) demonstrated high correlations only between DVR and DVR*R_1_ (range *r:*0.70–0.91). Comparisons with the dependent variables showed higher correlations between DVR and SUVR or SUVR_bias_ (range *r*:0.91–0.97, 0.24–0.60, respectively) than between DVR*R_1_ and SUVR or SUVR_bias_ (range *r*:0.57–0.87, 0.14–0.46, respectively). Therefore, GLMs were constructed without interaction terms, which resulted in the following models (1a and b). Note, the covariate “centre” is only included for [^18^F]flutemetamol.

1a $${\text{SUVR}} \sim {\text{DVR}} + R_{1} + {\text{Age}} + {\text{Sex}} + {\text{APOE}} - \varepsilon 4 + {\text{centre}}$$

1b $${\text{SUVR}}_{{{\text{bias}}}} \sim {\text{DVR}} + R_{1} + {\text{Age}} + {\text{Sex}} + {\text{APOE}} - \varepsilon 4 + {\text{centre}}$$

#### Relationship between SUVR and relative CBF

**Fig. 3:**
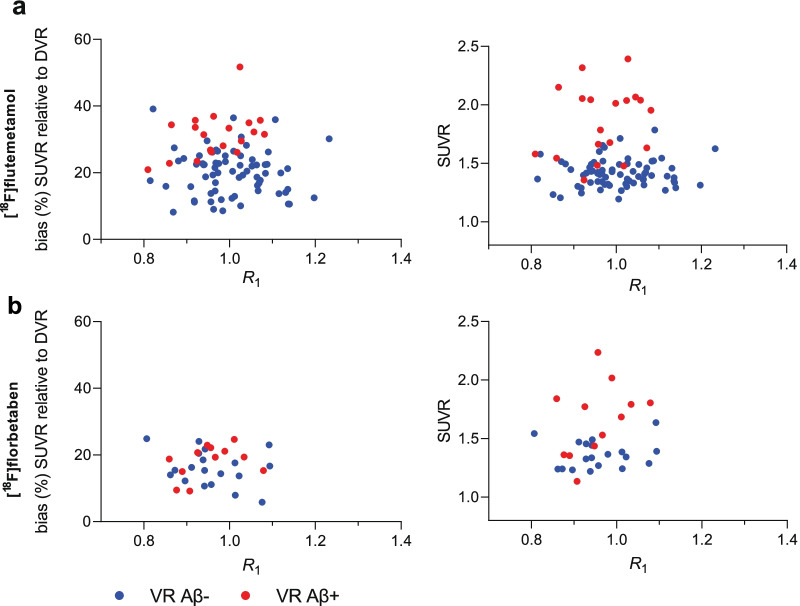
**Relationship between global cortical relative tracer delivery (*R***_**1**_**) and amyloid burden**. Correlation plots shown for **a** [^18^F]flutemetamol and **b** [^18^F]florbetaben. VR: visual read

As expected, there was a significant relationship between SUVR and DVR for both tracers across regions (range coefficient estimate [^18^F]flutemetamol: 1.60–1.84, [^18^F]florbetaben: 1.31–1.43, *p* < 0.001, Tables 2 and 3, Additional file 1: Table S2a-f). In addition, for both tracers there were negative associations between SUVR and *R*_1_ for the GCA (coefficient estimate [^18^F]flutemetamol: − 0.20, [^18^F]florbetaben: − 0.17, Fig. 3), PCC (coefficient estimate [^18^F]flutemetamol: − 0.20, [^18^F]florbetaben: − 0.21) and, in the case of [^18^F]flutemetamol, for the precuneus (coefficient estimate: − 0.20), albeit not statistically significant (*ns*) (Tables 2 and 3, Additional file 1: Table S2a,b,d). For [^18^F]flutemetamol specifically, a statistically significant association was also observed between SUVR and centre UNIGE, for the GCA and OFC (coefficient estimate: − 0.13 and 0.16, for both regions, respectively, *p* < 0.05, Table 2, Additional file 1: Table S2c). No statistically significant associations were observed between SUVR and the other independent variables.

**Table 2:** Relationship between (bias in) SUVR and independent variables for [^18^F]flutemetamol

GCA	SUVR		SUVR_bias_ (%)	
	Coefficient estimate	95% Confidence interval	Coefficient estimate (%)	95% Confidence interval (%)
DVR	1.70^‡^	1.55 to 1.85	32.04^‡^	19.78 to 44.31
*R*_1_	− 0.20	− 0.43 to 0.04	− 14.67	− 34.15 to 4.81
Age	0.00	0.00 to 0.01	0.17	− 0.10 to 0.44
Sex	0.00	− 0.04 to 0.03	− 0.18	− 3.17 to 2.81
*APOE*-ε4	0.03	− 0.01 to 0.07	2.72	− 0.30 to 5.75
BBRC	0.00	− 0.07 to 0.08	0.33	− 6.24 to 6.89
Amsterdam UMC	− 0.08	− 0.16 to 0.00	− 6.30	− 13.20 to 0.59
UNIGE	− 0.13*	− 0.24 to − 0.02	− 10.15*	19.17 to − 1.13

Females, *APOE*-ε4 non-carriers and centre UEDIN were used as reference groups

GCA: global cortical average, *p < 0.05, ^†^*p* < 0.01, ^‡^*p* < 0.001

**Table 3:** Relationship between (bias in) SUVR and independent variables for [^18^F]florbetaben

GCA	SUVR		SUVR_bias_ (%)	
	Coefficient estimate	95% Confidence interval	Coefficient estimate (%)	95% Confidence interval (%)
DVR	1.31^‡^	1.19 to 1.44	9.46	− 0.78 to 19.69
*R*_1_	− 0.17	− 0.49 to 0.14	− 13.43	− 39.00 to 12.14
Age	0.00	0.00 to 0.00	0.10	− 0.16 to 0.37
Sex	0.00	− 0.05 to 0.05	− 0.12	− 4.19 to 3.95
*APOE*-ε4	0.02	− 0.03 to 0.07	2.00	− 1.96 to 5.97

Females and *APOE*-ε4 non-carriers were used as reference groups

GCA: global cortical average, *p < 0.05, **p < 0.01, ^‡^*p* < 0.001

#### Relationship between bias in SUVR and relative CBF

For both tracers, positive associations were reported between SUVR_bias_ and DVR across regions (range coefficient estimate [^18^F]flutemetamol: 13.54–33.49%, *p* < 0.05 and [^18^F]florbetaben: 9.46–17.94%, statistically significant only for the precuneus *p* < 0.05, Tables 2 and 3, Additional file 1: Table S2a-f). In addition, negative associations were observed between SUVR_bias_ and *R*_1_ for the GCA (coefficient estimate [^18^F]flutemetamol: − 14.63%, [^18^F]florbetaben: − 13.43%, Fig. 3), PCC (coefficient estimate [^18^F]flutemetamol: − 9.99%, [^18^F]florbetaben: − 17.63%) and, in the case of [^18^F]flutemetamol, also for the precuneus and OFC (coefficient estimate: − 8.96% and − 6.77%, for both regions, respectively), albeit not statistically significant (Tables 2 and 3, Additional file 1: Table S2a-c,e). For [^18^F]flutemetamol, significant, positive associations were observed between SUVR_bias_ and *APOE*-ε4 (coefficient estimate precuneus: 3.30%, *p* < 0.05) and age (coefficient estimate PCC: 0.24%, *p* < 0.05), although with small coefficient estimates. Finally, associations between SUVR_bias_ and centre UNIGE and Amsterdam UMC were observed for the GCA (coefficient estimate: 6.32%, *ns* and − 10.16%, *p* < 0.05) and OFC (coefficient estimate: 5.99% and − 12.17%, *ns*), for both centres, respectively (Table 2, Additional file 1: Table S2c). No statistically significant associations were observed between SUVR_bias_ and the other independent variables.

## Discussion

The present study investigated whether bias in SUVR relative to DVR could be explained by factors such as underlying Aβ burden and relative CBF (as measured by *R*_1_). For both tracers, strong correlations were observed between SUVR and DVR, although SUVR overestimated DVR, and the magnitude of overestimation was positively associated with the degree of Aβ burden. Furthermore, negative associations were observed between SUVR or SUVR_bias_ and *R*_1_, albeit not statistically significant.

For both tracers, the observed proportional overestimation of SUVR relative to DVR is in line with previous work using [^11^C]PiB, where similar results in terms of overestimation and proportionality of bias were observed when comparing SUVR with DVR [15]. Carson and colleagues have mathematically demonstrated that target to reference tissue ratios (such as SUVR) overestimate true binding (such as DVR in the present work) at transient equilibrium conditions [3]. In particular, the magnitude of overestimation depends on tracer kinetics, with slower kinetics corresponding to larger overestimation. The present findings are in line with this work, given that a larger overestimation was observed for these Fluorine-18 tracers, as compared with [^11^C]PiB, which has faster kinetics [15, 30]. In addition, Carson and colleagues showed increased overestimation for high binding regions, as a result of the longer time required to reach equilibrium and the lower tissue clearance rates [3], which is in agreement with the present findings. Together, these findings suggest that in cross-sectional analyses including participants with low amyloid burden, the expected bias in global SUVR will be small, although in early binding regions it might already be substantial. Furthermore, in longitudinal analyses, caution is warranted as this proportional bias can falsely inflate SUVR-based accumulation rates [31]. Accordingly, in intervention trials, changes in SUVR-based accumulation rates may over- or underestimate the actual change in Aβ accumulation, leading to biased conclusions regarding the effectiveness of the drug or intervention. Overestimation of the overall signal (i.e. SUVR) might not be a major issue for anti-amyloid drugs with sufficiently large effects on Aβ pathology [32]; however, it could be of major concern when only small effects are expected, such as in lifestyle-related interventions [33]. In such cases, it would be advised to use quantitative instead of semi-quantitative measures of Aβ burden.

In mostly cognitively unimpaired individuals, there was no statistically significant effect of CBF on SUVR_bias_, although a negative association was observed between SUVR or SUVR_bias_ and CBF, as measured with *R*_1_. These findings appear to be in disagreement with a longitudinal [^11^C]PiB study of van Berckel and colleagues, which reported decreases in global *R*_1_ and concomitant decreases in global SUVR [4]. The discrepancy with the present study is likely due to differences in the study populations included. In the present study, the vast majority of participants were cognitively unimpaired, while in the study of van Berckel et al., these findings were related only to patients with AD dementia. It is known that large changes in CBF primarily occur at later disease stages, which could explain the association between *R*_1_ and SUVR in AD dementia patients in the study of van Berckel et al. and the lack of significant association in mainly cognitively unimpaired participants in the present study [7]. Interestingly, most regions showed a negative association between *R*_1_ and SUVR or SUVR_bias_ in this study, as would be expected with the progression of AD over time [7, 34, 35]. Furthermore, these findings demonstrate that changes in CBF are still small in mostly CU participants and that the effect on bias in SUVR is limited. However, this might not necessarily be the case when following these elderly individuals over time into advanced disease phases or when investigating disease-modifying drugs with unknown effects on CBF. In those scenarios, first quantitative measures should be acquired in at least a sub-sample of participants (e.g. in mono-centre Phase I and II studies) to understand whether changes in CBF play a role, before continuing with larger Phase II/III studies.

It should be noted that there were some between-tracer differences in terms of the relationship between SUVR and DVR (e.g. the association between global cortical SUVR and DVR was *R*^2^ = 0.85 for [^18^F]flutemetamol and *R*^2^ = 0.95 for [^18^F]florbetaben) and the association between *R*_1_ and SUVR or SUVR_bias_. Nevertheless, differences in the relationship between SUVR and DVR appeared to be smaller for the early ROIs. These between-tracer differences may be partially explained by differences in tracer kinetics, related to tracer clearance, degree of non-specific binding, or adherence to reference tissue assumptions [8, 9]. In addition, differences in demographic factors such as the percentage of Aβ-positive participants, age, sex, or *APOE*-ε4 carriers, also exerted an effect on the observed relationship between SUVR and DVR, as illustrated by the GLM analyses [15]. Furthermore, differences in these variables and the number of scanned participants per institute likely also explain the significant association between centre and SUVR or SUVR_bias_ for some regions (Additional file 1: Table S3). It is important to note that the large difference in sample size between the two tracer cohorts possibly also played a role; the [^18^F]flutemetamol cohort consisted of almost three times as many participants (*N* = 90) compared with the [^18^F]florbetaben cohort (*N* = 31). In addition, a limitation of the present work is that it investigated only cross-sectional relationships between SUVR, DVR, and *R*_1_, as no longitudinal data were available. However, longitudinal data are currently being collected in these subjects. Thus, in the future, it would be interesting to determine the magnitude of change in *R*_1_ over time in early AD stages and assess the relationship with bias in SUVR relative to DVR. Finally, it is important to note that the contribution of tracer clearance from plasma on (bias in) SUVR could not be tested, given that no (metabolite corrected) plasma input data were available in this study.

## Conclusion

The present findings show that the degree of bias in SUVR relative to DVR in mostly cognitively unimpaired individuals is directly related to the extent of underlying Aβ burden for both [^18^F]flutemetamol and [^18^F]florbetaben, which may be particularly problematic for longitudinal study designs. Furthermore, there were no statistically significant associations between *R*_1_ and bias in SUVR relative to DVR, likely because changes in CBF are still limited in participants with early Aβ pathology.

## Supplementary Information

**Additional file 1**. Impact of cerebral blood flow and amyloid load on SUVR bias.

## Data Availability

Data used in this study are not openly available at this stage due to ongoing data collection in the study. However, these will be made available to the research community upon end of the project (AMYPAD Prognostic and Natural History Study).

## Abbreviations

Aβ: Amyloid-beta
AD: Alzheimer’s disease
AMYPAD: AMYloid Imaging to Prevent Alzheimer’s Disease
*BP*_ND_: Non-displaceable binding potential
CBF: Cerebral blood flow
DVR: Distribution volume ratio
18F: Fluorine-18
MMSE: Mini-mental state examination
OFC: Orbitofrontal cortex
PCC: Posterior cingulate cortex
PET: Positron emission tomography
PNHS: Prognostic and Natural History Study
QC: Quality control
ROI: Region of interest
RPM: Receptor parametric mapping
SRTM: Simplified reference tissue method
SUVR: Standardized uptake value ratio
TAC: Time activity curve
2T4k_V_b_: Reversible two tissue compartment model (4-rate constants) with additional blood volume fraction parameter

## References

[1] Vizamyl [Internet]. [cited 2021 May 11]. Available from: https://www.gehealthcare.co.uk/en/products/categories/nuclear_imaging_agents/vizamyl

[2] Neuraceq [Internet]. Available from: https://www.ema.europa.eu/en/documents/product-information/neuraceq-epar-product-information_en.pdf

[3] Carson RE, Channing MA, Blasberg RG, Dunn BB, Cohen RM, Rice KC (1993). Comparison of bolus and infusion methods for receptor quantitation: application to [18 F]cyclofoxy and positron emission tomography. J Cereb Blood Flow Metab.

[4] van Berckel BNM, Ossenkoppele R, Tolboom N, Yaqub M, Foster-Dingley JC, Windhorst AD (2013). Longitudinal amyloid imaging using 11C-PiB: methodologic considerations. J Nucl Med.

[5] Collij LE, Konijnenberg E, Reimand J, ten Kate M, den Braber A, Alves IL (2019). Assessing amyloid pathology in cognitively normal subjects using ^18^ F-flutemetamol PET: comparing visual reads and quantitative methods. J Nucl Med.

[6] Lammertsma AA (2017). Forward to the past: the case for quantitative PET imaging. J Nucl Med.

[7] Jack CR, Knopman DS, Jagust WJ, Petersen RC, Weiner MW, Aisen PS (2013). Update on hypothetical model of Alzheimer’s disease biomarkers. Lancet Neurol.

[8] Heurling K, Buckley C, Van Laere K, Vandenberghe R, Lubberink M (2015). Parametric imaging and quantitative analysis of the PET amyloid ligand [(18)F]flutemetamol. Neuroimage.

[9] Becker GA, Ichise M, Barthel H, Luthardt J, Patt M, Seese A (2013). PET quantification of 18F-florbetaben binding to β-amyloid deposits in human brains. J Nucl Med.

[10] Sakr FA, Grothe MJ, Cavedo E, Jelistratova I, Habert M-O, Dyrba M (2019). Applicability of in vivo staging of regional amyloid burden in a cognitively normal cohort with subjective memory complaints: the INSIGHT-preAD study. Alzheimer’s Res Ther.

[11] Guo T, Landau SM, Jagust WJ, Alzheimer’s Disease Neuroimaging Initiative. Detecting earlier stages of amyloid deposition using PET in cognitively normal elderly adults. Neurology. 2020;94:e1512–24. 10.1212/WNL.0000000000009216 PMC7251521 32188766

[12] Collij LE, Heeman F, Salvadó G, Ingala S, Altomare D, Wilde A (2020). Multi-tracer model for staging cortical amyloid deposition using PET imaging. Neurology.

[13] Binnewijzend MAA, Benedictus MR, Kuijer JPA, van der Flier WM, Teunissen CE, Prins ND (2016). Cerebral perfusion in the predementia stages of Alzheimer’s disease. Eur Radiol.

[14] Sojkova J, Beason-Held L, Zhou Y, An Y, Kraut MA, Ye W (2008). Longitudinal cerebral blood flow and amyloid deposition: an emerging pattern?. J Nucl Med.

[15] Lopes Alves I, Collij LE, Altomare D, Frisoni GB, Saint-Aubert L, Payoux P (2020). Quantitative amyloid PET in Alzheimer’s disease: the AMYPAD prognostic and natural history study. Alzheimer’s Dement.

[16] Heeman F, Yaqub M, Lopes Alves I, Heurling K, Berkhof J, Gispert JD (2019). Optimized dual-time-window protocols for quantitative [18F]flutemetamol and [18F]florbetaben PET studies. EJNMMI Res.

[17] Klein S, Staring M, Murphy K, Viergever MA, Pluim JPW (2010). elastix: a toolbox for intensity-based medical image registration. IEEE Trans Med Imaging.

[18] Shamonin DP, Bron EE, Lelieveldt BPF, Smits M, Klein S, Staring M (2014). Fast parallel image registration on CPU and GPU for diagnostic classification of Alzheimer’s Disease. Front Neuroinform.

[19] Wolz R, Aljabar P, Hajnal JV, Hammers A, Rueckert DLEAP (2010). Learning embeddings for atlas propagation. Neuroimage.

[20] Wu Y, Carson RE (2002). Noise reduction in the simplified reference tissue model for neuroreceptor functional imaging. J Cereb Blood Flow Metab.

[21] Boellaard R, Yaqub M, Lubberink M, Lammertsma A. PPET: A software tool for kinetic and parametric analyses of dynamic PET studies. NeuroImage. 2006;Supplement 2:T62.

[22] Heeman F, Yaqub M, Hendriks J, Bader I, Barkhof F, Gispert JD (2021). Parametric imaging of dual-time window [18F]flutemetamol and [18F]florbetaben studies. Neuroimage.

[23] Gunn RN, Lammertsma AA, Hume SP, Cunningham VJ (1997). Parametric imaging of ligand-receptor binding in PET using a simplified reference region model. Neuroimage.

[24] Peretti DE, Reesink FE, Doorduin J, de Jong BM, De Deyn PP, Dierckx RAJO (2019). Optimization of the k2′ parameter estimation for the pharmacokinetic modeling of dynamic PIB PET scans using SRTM2. Front Phys.

[25] Klunk WE, Koeppe RA, Price JC, Benzinger TL, Devous MD, Jagust WJ (2015). The Centiloid Project: standardizing quantitative amyloid plaque estimation by PET. Alzheimer’s Dementia.

[26] Insel PS, Mormino EC, Aisen PS, Thompson WK, Donohue MC (2020). Neuroanatomical spread of amyloid β and tau in Alzheimer’s disease: implications for primary prevention. Brain Commun.

[27] Mattsson N, Palmqvist S, Stomrud E, Vogel J, Hansson O. Staging β-amyloid pathology with amyloid positron emission tomography. JAMA Neurol. 2019. 10.1001/jamaneurol.2019.2214 PMC6646987 31314895

[28] Martin Bland J, Altman Douglas G (1986). Statistical methods for assessing agreement between two methods of clinical measurement. Lancet.

[29] Mukaka M (2012). A guide to appropriate use of Correlation coefficient in medical research. Malawi Med J.

[30] Klunk WE, Engler H, Nordberg A, Wang Y, Blomqvist G, Holt DP (2004). Imaging brain amyloid in Alzheimer’s disease with Pittsburgh Compound-B. Ann Neurol.

[31] Lopes Alves I, Heeman F, Collij LE, Salvadó G, Tolboom N, Vilor-Tejedor N (2021). Strategies to reduce sample sizes in Alzheimer’s disease primary and secondary prevention trials using longitudinal amyloid PET imaging. Alzheimers Res Ther.

[32] Sevigny J, Chiao P, Bussière T, Weinreb PH, Williams L, Maier M (2016). The antibody aducanumab reduces Aβ plaques in Alzheimer’s disease. Nature.

[33] Rosenberg A, Mangialasche F, Ngandu T, Solomon A, Kivipelto M (2020). Multidomain interventions to prevent cognitive impairment, alzheimer’s disease, and dementia: from FINGER to world-wide FINGERS. J Prev Alzheimers Dis.

[34] Duan W, Sehrawat P, Balachandrasekaran A, Bhumkar AB, Boraste PB, Becker JT (2020). Cerebral blood flow is associated with diagnostic class and cognitive decline in Alzheimer’s disease. J Alzheimers Dis.

[35] Matsuda H (2001). Cerebral blood flow and metabolic abnormalities in Alzheimer’s disease. Ann Nucl Med.

