# Supplementary material for: Impact of cerebral blood flow and amyloid load on SUVR bias
Source: EJNMMI Res. 2022 May 12;12:29. doi: 10.1186/s13550-022-00898-8 (PMC9098761; doi:10.1186/s13550-022-00898-8)
Supplement: Supplementary file 1 — Additional file 1. Impact of cerebral blood flow and amyloid load on SUVR bias. [file 13550_2022_898_MOESM1_ESM.docx]

**Supplementary materials**

**Impact of cerebral blood flow and amyloid load on suvr bias**

Fiona Heeman^1^, Maqsood Yaqub^1^, Janine Hendriks^1^, Bart N.M. van Berckel^1^, Lyduine E. Collij^1^, Katherine R. Gray^2^ , Richard Manber^2^, Robin Wolz^2^, Valentina Garibotto^3,4^, Catriona Wimberley^5^, Craig Ritchie^5^, Frederik Barkhof^1,6,^, Juan Domingo Gispert^,7-10^, David Vállez García^1^, Isadora Lopes Alves^1^, Adriaan A. Lammertsma^1^, *on behalf of the AMYPAD Consortium*

**Corresponding author:**

Fiona Heeman, ORCID:0000-0001-7769-8329

Amsterdam UMC, VUmc, Radiology and Nuclear Medicine, De Boelelaan 1117, 1081 HV Amsterdam, Netherlands

E:[f.heeman@amsterdamumc.nl](mailto:f.heeman@amsterdamumc.nl) T:+31(0)20 444 4837

^1^Amsterdam UMC, Vrije Universiteit Amsterdam, Radiology and Nuclear Medicine, Amsterdam Neuroscience, De Boelelaan 1117, Amsterdam, Netherlands

^2^IXICO Plc, London, United Kingdom.

^3^NIMTLab, Faculty of Medicine, Geneva University, Geneva, Switzerland.

^4^Division of Nuclear Medicine and Molecular Imaging, Geneva University Hospitals, Geneva, Switzerland.

^5^Edinburgh Imaging, Queen's Medical Research Institute, University of Edinburgh, Edinburgh, UK

^6^UCL, Institutes of Neurology and Healthcare Engineering, London,United Kingdom

^7^Barcelonaβeta Brain Research Centre, Pasqual Maragall Foundation, Barcelona, Spain

^8^Centro de Investigación Biomédica en Red de Bioingeniería, Biomateriales y Nanomedicina (CIBER-BBN), Madrid, Spain

^9^Department of Experimental and Health Sciences, Universitat Pompeu Fabra, Barcelona

^10^IMIM (Hospital del Mar Medical Research Institute), Barcelona, Spain

| **Supplementary Table 1. Acquisition methods per centre** | | | | | |
| --- | --- | --- | --- | --- | --- |
|  | **UEDIN** | **BBRC** | **Amsterdam UMC, VUmc** | **UNIGE** |  |
| **Scanner manufacturer** | Siemens | Siemens | Philips | Siemens |  |
| **Scanner model** | Biograph mMR | Biograph64 mCT | Ingenuity TF PET/MR | Biograph128 mCT |  |
| **Pixel Spacing** | 1.043*1.043 | 1.0182*1.018 | 2.000*2.000 | 1.018*1.018 |  |
| **Reconstruction method** | OP-OSEM4i21s | OSEM3D 4i24s | LOR-RAMLA | OSEM3D 4i24s |  |
| **Attenuation correction** | MR-based, HiRes | CT-based | MR-based | CT-based |  |
| **Scatter correction** | Model based, relative, single scatter simulation | Model-based, relative, single scatter simulation | SS-SIMUL | Model-based, relative, single scatter simulation |  |

Amsterdam UMC, VUmc = Amsterdam University Medical Centre, Vrije Universiteit medical center BBRC = Barcelona Beta Brain Research Center, UNIGE= University of Geneva, UEDIN= The University of Edinburgh. OSEM: ordered-subset expectation maximization, LOR-RAMLA: standard line-of-response–based row-action maximum-likelihood algorithm

**Supplementary Table 2a.**

**Relationship between (bias in) SUVR and independent variables for [^18^F]flutemetamol**

| **Precuneus** | **SUVR** | | **SUVR_bias_ (%)** | |
| --- | --- | --- | --- | --- |
|  | **Coefficient estimate** | **95% Confidence interval** | **Coefficient estimate (%)** | **95% Confidence interval (%)** |
| **DVR** | 1.84^†^ | 1.66 – 2.03 | 33.55^†^ | 21.58 – 45.53 |
| ***R*_1_** | -0.20 | -0.43 - 0.03 | -8.83 | -23.86 – 6.20 |
| **Age** | 0.00 | 0.00 - 0.01 | 0.23 | -0.01– 0.47 |
| **Sex** | -0.01 | -0.06 - 0.03 | -0.35 | -3.09 - 2.38 |
| ***APOE-*ɛ4** | 0.05* | -0.01 - 0.09 | 3.34* | -0.58 – 6.09 |
| **BBRC** | -0.05 | -0.14 - 0.04 | -3.81 | -9.77 – 2.14 |
| **Amsterdam UMC** | -0.04 | -0.14 - 0.05 | -2.45 | -8.71 – 3.81 |
| **UNIGE** | -0.03 | -0.15 - 0.10 | -3.40 | -11.54 - 4.75 |

Females, *APOE*-ɛ4 non-carriers and centre UEDIN were used as reference groups, **p*<0.05, ***p*<0.01, ^†^*p*<0.001

**Supplementary Table 2b.**

**Relationship between (bias in) SUVR and independent variables for [^18^F]flutemetamol**

| **PCC** | **SUVR** | | **SUVR_bias_(%)** | |
| --- | --- | --- | --- | --- |
|  | **Coefficient estimate** | **95% Confidence interval** | **Coefficient estimate (%)** | **95% Confidence interval (%)** |
| **DVR** | 1.60^†^ | 1.42 - 1.79 | 13.69* | 3.23 – 24.16 |
| ***R*_1_** | -0.20 | -0.44 - 0.04 | -9.38 | -22.70 – 3.94 |
| **Age** | 0.00 | 0.00 - 0.01 | 0.24* | 0.02 – 0.45 |
| **Sex** | -0.01 | -0.06 - 0.03 | -0.64 | -2.99 – 1.70 |
| ***APOE-*ɛ4** | 0.03 | -0.01 - 0.07 | 1.46 | -0.86 – 3.78 |
| **BBRC** | 0.00 | -0.09 - 0.09 | -1.87 | -6.89 – 3.15 |
| **Amsterdam UMC** | 0.03 | -0.07 - 0.12 | 0.29 | -4.94– 5.52 |
| **UNIGE** | 0.06 | -0.07 - 0.18 | 1.66 | -5.32 – 8.64 |

Females, *APOE*-ɛ4 non-carriers and centre UEDIN were used as reference groups, PCC: posterior cingulate

cortex, **p*<0.05, ***p*<0.01, ^†^*p*<0.001

**Supplementary Table 2c.**

**Relationship between (bias in) SUVR and independent variables for [^18^F]flutemetamol**

| **OFC** | **SUVR** | | **SUVR_bias_(%)** | |
| --- | --- | --- | --- | --- |
|  | **Coefficient estimate** | **95% Confidence interval** | **Coefficient estimate (%)** | **95% Confidence interval (%)** |
| **DVR** | 1.60^†^ | 1.42 - 1.79 | 25.06** | 8.99 – 41.12 |
| ***R*_1_** | 0.02 | -0.31 - 0.35 | -6.78 | -21.27 – 34.83 |
| **Age** | 0.00 | 0.00 - 0.01 | 0.34 | -0.04 – 0.71 |
| **Sex** | -0.02 | -0.06 - 0.03 | -1.84 | -5.79 - 2.11 |
| ***APOE-*ɛ4** | 0.01 | -0.04 - 0.06 | 1.10 | -2.90 – 5.11 |
| **BBRC** | 0.03 | -0.07 - 0.14 | 2.99 | -5.79 – 11.77 |
| **Amsterdam UMC** | 0.06 | -0.05 - 0.17 | 6.00 | -3.36 – 15.35 |
| **UNIGE** | 0.16* | 0.01 - 0.30 | -12.17 | -0.15 – 24.50 |

Females, *APOE*-ɛ4 non-carriers and centre UEDIN were used as reference groups, OFG: orbitofrontal gyrus,

**p*<0.05, ***p*<0.01, ^†^*p*<0.001

**Supplementary Table 2d.**

**Relationship between (bias in) SUVR and independent variables for [^18^F]florbetaben**

| **Precuneus** | **SUVR** | | **SUVR_bias_(%)** | | |
| --- | --- | --- | --- | --- | --- |
|  | **Coefficient estimate** | **95% Confidence interval** | **Coefficient estimate (%)** | | **95% Confidence interval (%)** |
| **DVR** | 1.41^†^ | 1.27 - 1.55 | 17.94^†^ | 5.78 – 30.11 | |
| ***R*_1_** | -0.05 | -0.35 - 0.25 | -1.83 | -27.45 - 23.80 | |
| **Age** | 0.00 | 0.00 - 0.01 | 0.32 | 0.00- 0.64 | |
| **Sex** | -0.06 | -0.11 - 0.00 | -4.54 | -9.42 - 0.35 | |
| ***APOE-*ɛ4** | 0.02 | -0.03 - 0.08 | 2.00 | -2.77 – 6.78 | |

Females and *APOE*-ɛ4 non-carriers were used as reference groups. **p*<0.05, ***p*<0.01, ^†^*p*<0.001

**Supplementary Table 2e.**

**Relationship between (bias in) SUVR and independent variables for [^18^F]florbetaben**

| **PCC** | **SUVR** | | **SUVR_bias_(%)** | | |
| --- | --- | --- | --- | --- | --- |
|  | **Coefficient estimate** | **95% Confidence interval** | **Coefficient estimate (%)** | | **95% Confidence interval (%)** |
| **DVR** | 1.43† | 1.21 - 1.65 | 15.14 | -3.79 – 34.07 | |
| ***R*_1_** | -0.21 | -0.59 - 0.17 | -17.63 | -50.15 – 14.90 | |
| **Age** | 0.00 | 0.00 - 0.01 | 0.32 | -0.07- 0.71 | |
| **Sex** | -0.05 | -0.12 - 0.01 | -4.57 | -10.55 – 1.41 | |
| ***APOE-*ɛ4** | 0.06 | -0.01 - 0.12 | 4.75 | -0.94 – 10.43 | |

Females and *APOE*-ɛ4 non-carriers were used as reference groups, PCC: posterior cingulate cortex,

**p*<0.05, ***p*<0.01, ^†^*p*<0.001

**Supplementary Table 2f.**

**Relationship between (bias in) SUVR and independent variables for [^18^F]florbetaben**

| **OFC** | **SUVR** | | **SUVR_bias_(%)** | | |
| --- | --- | --- | --- | --- | --- |
|  | **Coefficient estimate** | **95% Confidence interval** | **Coefficient estimate (%)** | | **95% Confidence interval (%)** |
| **DVR** | 1.37^†^ | 1.15 - 1.59 | 16.74 | -1.87 – 35.36 | |
| ***R*_1_** | 0.01 | -0.46 - 0.47 | 1.85 | -37.01 - 40.70 | |
| **Age** | 0.00 | -0.01 - 0.00 | -0.13 | -0.56 - 0.31 | |
| **Sex** | -0.05 | -0.13 - 0.03 | -4.36 | -10.92 – 2.20 | |
| ***APOE-*ɛ4** | -0.01 | -0.08 - 0.06 | -0.45 | -6.60 - 5.69 | |

Females and *APOE*-ɛ4 non-carriers were used as reference groups, OFG: orbitofrontal gyrus,

**p*<0.05, ***p*<0.01, ^†^*p*<0.001

|  | **Supplementary Table 3. Demographic differences between centres** | | | | | | | |
| --- | --- | --- | --- | --- | --- | --- | --- | --- |
| **Centre** | | **# Participants** | **Age** | **Females (%)** | **MMSE** | ***APOE*-ɛ4**  **Carriers (%)** | **DVR** | ***R*_1_** |
| **UEDIN** | | 5 | 62.6 | 40.0 | 29.2 | 60.0 | 1.16 | 1.07 |
| **BBRC** | | 29 | 64.7 | 51.7 | 29.0 | 27.6 | 1.15 | 1.03 |
| **Amsterdam UMC** | | 51 | 68.7 | 54.9 | 29.0 | 45.1 | 1.26 | 0.97 |
| **UNIGE** | | 5 | 69.6 | 80.0 | 27.4 | 60.0 | 1.40 | 1.03 |

MMSE: mini-mental state examination
